# “I Want to Create So Much Stimulus That Adaptation Goes Through the Roof”: High-Performance Strength Coaches' Perceptions of Planned Overreaching

**DOI:** 10.3389/fspor.2022.893581

**Published:** 2022-05-02

**Authors:** Lee Bell, Alan Ruddock, Tom Maden-Wilkinson, David Rogerson

**Affiliations:** Department of Sport and Physical Activity, Sheffield Hallam University, Sheffield, United Kingdom

**Keywords:** overreaching, functional overreaching, overtraining, strength sports, strength training

## Abstract

Functional overreaching (FOR) occurs when athletes experience improved athletic capabilities in the days and weeks following short-term periods of increased training demand. However, prolonged high training demand with insufficient recovery may also lead to non-functional overreaching (NFOR) or the overtraining syndrome (OTS). The aim of this research was to explore strength coaches' perceptions and experiences of planned overreaching (POR); short-term periods of increased training demand designed to improve athletic performance. Fourteen high-performance strength coaches (weightlifting; *n* = 5, powerlifting; *n* = 4, sprinting; *n* = 2, throws; *n* = 2, jumps; *n* = 1) participated in semistructured interviews. Reflexive thematic analysis identified 3 themes: *creating enough challenge, training prescription*, and *questioning the risk to reward*. POR was implemented for a 7 to 14 day training cycle and facilitated through increased daily/weekly training volume and/or training intensity. Participants implemented POR in the weeks (~5–8 weeks) preceding competition to allow sufficient time for performance restoration and improvement to occur. Short-term decreased performance capacity, both during and in the days to weeks following training, was an anticipated by-product of POR, and at times used as a benchmark to confirm that training demand was sufficiently challenging. Some participants chose not to implement POR due to a lack of knowledge, confidence, and/or perceived increased risk of athlete training maladaptation. Additionally, this research highlights the potential dichotomy between POR protocols used by strength coaches to enhance athletic performance and those used for the purpose of inducing training maladaptation for diagnostic identification.

## Introduction

Optimal performance in strength sports is achieved through careful manipulation of training and recovery and facilitated through strategic resistance exercise programming relative to competition schedule (Storey and Smith, 2012). To invoke the physiological adaptations necessary to achieve a meaningful standard of performance, the training process must provide an appropriate stimulus without training maladaptation (DeWeese et al., 2015a). In strength sports such as weightlifting, powerlifting, and maximal effort throws, short-term periods of increased training demand have been reported to improve characteristics that contribute to optimal performance, such as maximal strength, impulsiveness, and rate of force development (Pistilli et al., 2008; Zourdos et al., 2016; Bazyler et al., 2017, 2018; Travis et al., 2020a). These short-term, concentrated “mini preparation” training cycles have been referred to as planned overreaching (POR), or simply “overreaching” in the literature (Pistilli et al., 2008; Meeusen et al., 2013; Stone et al., 2021). POR is typically implemented into the athlete's training programme through a deliberate and often dramatic increase in training volume, facilitated via multiple daily training sessions and/or training intensity (Pistilli et al., 2008; Storey and Smith, 2012; Travis et al., 2020b). Moreover, POR is often undertaken during competition and/or peaking phases of a training schedule for several days (7–14 days), separated by longer periods of normal training or tapering to reduce the risk of maladaptation (Pistilli et al., 2008; Travis et al., 2020b; Stone et al., 2021).

The objective of POR is to achieve functional overreaching (FOR) which is characterized by performance improvement above the initial baseline (DeWeese et al., 2015b), observed only after an initial period (2–5 weeks) of performance decline from baseline (Pistilli et al., 2008; Kreher and Schwartz, 2012; Meeusen et al., 2013; DeWeese et al., 2015b). Non-functional overreaching (NFOR) is characterized by impaired performance lasting several days to weeks, with no performance improvement above the initial baseline (Halson and Jeukendrup, 2004; Meeusen et al., 2013). During prolonged or excessive training without sufficient recovery, the overtraining syndrome (OTS) may occur (Meeusen et al., 2013). The OTS is characterized by a long-term reduction in performance lasting several weeks to months (Meeusen et al., 2013). To date, no single test or assessment has been developed that can reliably detect the transitory point where periods of increased training demand such as POR result in either FOR or NFOR/OTS, making it difficult for coaches to identify optimal training demand to achieve FOR and avoid maladaptive states such as NFOR/OTS (Fry and Kraemer, 1997; Bell et al., 2020; Grandou et al., 2020b). The latest consensus, in the scientific community, suggests that OTS and NFOR can only be differentiated by retrospective recovery time-course, and not the type of training stress, the magnitude of impairment, or profile of symptoms (Meeusen et al., 2013).

Previous research exploring the effects of POR in strength athlete populations has focused largely on prospective cohort (Warren et al., 1992; Fry et al., 1993, 2000a; Hartman et al., 2007; Haff et al., 2008; Bazyler et al., 2017; Khlif et al., 2019; Suarez et al., 2019) and longitudinal observational studies involving weightlifting athletes (Häkkinen et al., 1987, 1989; Fry et al., 1994b), as well as case studies involving both weightlifting (Bazyler et al., 2018; Travis et al., 2020a) and maximal effort throws athletes (Bazyler et al., 2017). These study designs facilitate the assessment of exposure to tailored POR protocols, as well as the analysis of baseline data at different time points with or without manipulation of the training environment. However, it can be difficult to ensure consistent assessment of participants at each time point during the research, especially during observational research. Moreover, the control of confounding variables can also be a challenge. Therefore, whilst these studies provide evidence for the potential causative inference between undertaking POR and the resulting performance, they cannot prove causality (Sedgwick, 2013).

Improved sport-specific performance (i.e., weightlifting, throws) and/or general measures of athletic performance indicative of FOR (i.e., maximal strength) has been reported in some studies utilizing POR (Häkkinen et al., 1989; Warren et al., 1992; Fry et al., 1993, 2000a; Pistilli et al., 2008; Bazyler et al., 2017; Suarez et al., 2019). However, performance plateau or NFOR has been reported in others (Fry et al., 1994c,d, 1998, 2006; Purdom et al., 2021). Overall, the number of studies reporting performance improvement after a period of high training demand (i.e., FOR) outweigh those that have observed NFOR (Bell et al., 2020; Grandou et al., 2020b). There is only minimal evidence that true OTS has occurred in either competitive strength athletes or in athletes undertaking resistance-based exercise (Bell et al., 2020; Grandou et al., 2020a,b). Moreover, high-performance strength coaches perceive both the risk and prevalence of OTS within their sport to be low (Bell et al., 2021).

In high-performance strength sport, periodisation is often viewed as the “gold standard” approach to training theory, used to maximize physiological adaptations whilst simultaneously avoiding the OTS (Plisk and Stone, 2003). Although different models exist, a central tenet of periodisation is that training is divided into a number of focused phases of training, structured and designed to acheive peak performance at specific timepoints (Suchomel et al., 2018). Moreover, periodisation is built on the implicit assumption that the magnitude and time course of physiological adaptation can be predicted (Suchomel et al., 2018). Whilst there is evidence to suggest that systematic variation of training can lead to improvements in athletic performance, there is limited evidence to suggest that a superior framework of periodisation exists, or that periodisation is superior to non-periodised training (Kiely, 2018; Afonso et al., 2019; Kataoka et al., 2021). There is little agreement on a universally accepted definition of periodisation, and the term “periodisation” is often used interchangeably with “programming,” making it difficult to determine it efficacy against non-periodised approaches (Afonso et al., 2019; Kataoka et al., 2021). Further, when the physiological response to structured training is analyzed at an inter-individual level, athletes typically exhibit variability in training adaptations (Kiely, 2018), making it difficult for the coach to predict how athletes might adapt to structured training. There is a clear scarcity of research investigating periodised training, large heterogeneity between research studies, and a lack of studies investigating the accuracy of predicted adaptations that require further investigation. This presents a problem for strength coaches who intends to use structured periods of high training demand or seek the “best” periodisation framework to achieve FOR and avoid NFOR/OTS, as the specific response to such training cannot easily be predicted and is highly variable (Kiely, 2018; Afonso et al., 2019).

A lack of understanding of the terminology and conceptualization of OTS between expert consensus, sports science researchers and strength coaches have highlighted the need to develop evidence-informed collaboration between strength coaches and sports scientists (Bell et al., 2021). Without guidance from coaches and practitioners, research may not fully elucidate the complexity of the training response to POR, or the multidimensional dilemmas faced by strength coaches when working with high-performance athletes. Previous commentary has highlighted that the best coaches are often years ahead of sports science research when it comes to the prescription and supervision of individualized training (Haugen, 2021). However, research exploring the “secrets” of the athlete training process from the perspective of the coach within sports science literature is limited, and whilst there is an ever-increasing amount of empirical research dedicated to optimizing athlete training practices, there remains a considerable gap between science and good practice (Haugen, 2021; Haugen et al., 2021). As such, involving coaches in the development of knowledge relating to POR is fundamental to improved understanding. Therefore, this study aimed to explore high-performance strength coaches' perceptions of POR and to provide a new way of understanding and conceptualizing the prescription of POR in practice.

## Materials and Methods

### Approach to the Problem

A qualitative research design was adopted for this study as it enables the exploration of experiences arising from human behavior (Smith and Sparkes, 2016). A semistructured interview format was selected to provide a systematic but flexible framework of inquiry to ensure comprehensive information collection (Tenenbaum and Driscoll, 2005). Semistructured interviews are considered an appropriate qualitative research tool where perceptions and opinions of participants can be complex, nuanced, and encompass values, intentions, and ideals (Kallio et al., 2016). Effective semistructured interviews facilitate a dynamic and iterative interaction between interviewer and interviewee (DeJonckheere and Vaughn, 2019) and are designed to promote a deep exploration of participants' experiences and attitudes toward the topic of interest (Jamshed, 2014). Therefore, throughout each interview, participants were encouraged to draw upon their own experiences and to provide experiential responses. Data were analyzed using reflexive thematic analysis using guidelines provided by Braun and Clarke (2006), which facilitated the identification, organization, and subsequent analysis of qualitative data into meaningful patterns (Braun and Clarke, 2006; Nowell et al., 2017).

### Participants and Sampling

After ethical approval [ER16222001], volunteers provided informed consent to participate in the study which was conducted according to the 7th revision of the Declaration of Helsinki (World Medical Association, 2013). Fourteen high-performance strength coaches were recruited using an opportunity sampling approach. Participants represented a cross-section of coaches from strength sports: weightlifting; *n* = 5, powerlifting; *n* = 4, sprinting; *n* = 2, throws; *n* = 2, jumps; *n* = 1. Participants were considered high-performance strength coaches if they met the inclusion criteria of ≥3 years' experience of coaching to at least national standard in a strength sport (which were defined for the purpose of this research as weightlifting, powerlifting, sprinting, jumps [e.g., long jump, triple jump) or throws sports (e.g., hammer, discus, javelin)]. A descriptive profile of each participant is located in Table 1. Educational achievement ranged from high school qualifications to doctorate, with 6 participants possessing an undergraduate degree in a relevant subject area as their highest academic qualification, and 5 possessing a postgraduate degree in a related field. Participants held appropriate national governing body certifications, with most also in possession of a strength and conditioning accreditation (e.g., National Strength and Conditioning Association, United Kingdom Strength and Conditioning Association). The sample size deemed appropriate for this study was led by the principle of data saturation (Saunders et al., 2018). An initial non-probabilistic sample size of ≥6 participants was projected to achieve information redundancy and therefore fail to provide additional novel information (Guest et al., 2006). However, because data saturation is difficult to determine before analysis (Braun and Clarke, 2021), participants were continuously recruited until no new themes were identified and interviews failed to return new or novel information.

**Table 1:** Descriptive characteristics of participants.

**Participant identification number**	**Sport**	**Experience (years)**	**Experience level**
1	Powerlifting	15	International
2	Powerlifting	6	National
3	Weightlifting	4	International
4	Weightlifting	12	International
5	Powerlifting	10	International
6	Powerlifting	5	International
7	Weightlifting	20	International
8	Sprints	10	International
9	Jumps	13	International
10	Weightlifting	9	International
11	Throws	21	International
12	Weightlifting	57	International
13	Throws	15	International
14	Sprints	4	International

### Procedure

Interviews were collected by the principal investigator (L.B.) either online or face-to-face depending on geographical location and availability. Due to the exploratory nature of the research, a semistructured interview approach was chosen to facilitate flexible and in-depth information collection whilst remaining objective and focused on the research question (Kallio et al., 2016). An interview guide was developed by the principal investigator as part of a broader qualitative exploration into strength training practices in high-performance coaching and refined and adapted during pilot interviews. The full interview guide can be found in Appendix 1. During each interview, the lead investigator collected detailed field notes to act as prompts for further questions, and to ensure topics were explored in sufficient depth. Participants were encouraged to answer questions comprehensively, providing detailed experiences and examples. Online interviews were recorded using European Union General Data Protection Regulation-compliant software (Skype Ltd, version 15, Luxembourg). Face-to-face interviews were conducted in a mutually agreed, unobtrusive environment, and audio was captured using a digital voice recording device (Zoom, Hn1 digital voice recorder 2.0, UK). Interviews were transcribed verbatim and both audio recordings and transcripts from each interview were exported to a password-protected external hard drive (Seagate Technology PLC, Fremont, California, USA) for storage. Participants were randomly assigned an identification number between one and 14 (using a random number programme) so that personal information could be anonymised during publicizing of results.

### Reflexivity

The principal investigator of this research is a Senior Lecturer in sport and exercise science with both practical experience and research interest in strength sports, and who has previously published qualitative research using reflexive thematic analysis. The primary research question was developed as part of a wider investigation into the understanding of NFOR/OTS in strength sports from the perspective of the high-performance coach; a topic that lacks qualitative analysis.

In qualitative research, *reflexivity* is an integral aspect of transparency during qualitative research practice (Korstjens and Moser, 2018) and acknowledges how the relationship between researcher and participant might influence the construction of knowledge during the research process (Nyirenda et al., 2020). To enhance trustworthiness and reflexivity, the background of the principal investigator was made transparent to participants prior to each interview. Moreover, the principal investigator sought to remove any pre-conceived assumptions relating to the research topic and distinguish their own ideas and experiences from those held by participants to aid objectivity during information collection and analysis (Price and Martin, 2018). To strengthen the credibility, accuracy and trustworthiness of this research, an audit trail of notes made during each interview were maintained, as well as the development of a code book, and notes made during research team meetings.

### Data Analysis

Interview transcripts were exported to NVivo Pro (v11.4.1.1064, Flexera Software LLC; Itasca, IL, USA) and analyzed using reflexive thematic analysis as described by Braun and Clarke (2006). The first stage of analysis involved repeated listening to interview recordings, as well as reading of transcripts and field notes. During this stage, sections of text from each transcript were highlighted if they provided preliminary “points of interest” based on overall meaningfulness and relevance. These initial ideas were used to develop codes; labels assigned to aspects of the dataset that summarize important concepts and have relevance to the research question (Braun and Clarke, 2006; Nowell et al., 2017). Next, codes were organized into broad themes that helped to categorize important information related to the research question into meaningful patterns. Subthemes were developed to assist in organizing the large dataset into specific elements and to aid in the reporting of results (Braun and Clarke, 2006).

When research is aimed at informing practice, trustworthiness (a term used synonymously with reliability and validity within qualitative research), is an important step to ensure applicability of findings (Nowell et al., 2017; Roberts et al., 2019). To ensure trustworthiness, themes and subthemes were reviewed and refined throughout the data analysis process, in that they were updated, amended, deleted, or merged regularly as recommended by Braun and Clarke (2006). A codebook was created and updated by the principal investigator to facilitate reflexivity and objectivity, and to maintain an audit trail of data saturation (Guest et al., 2006; Braun and Clarke, 2020). To enhance methodological rigor, members of the research team each individually and blindly coded a sample transcript at the early stages of analysis, discussed their interpretation of data patterns and proposed themes during a research team meeting (Nowell et al., 2017). This process allowed scrutiny of data and an opportunity to consider alternative interpretations (Cutcliffe and McKenna, 1999). Additional research team meetings were organized at regular and important intervals during the analysis process, and written records were maintained to develop an audit trail of methodological decisions (Nowell et al., 2017). In the final stage of analysis, themes were confirmed by all members of the research team once it was determined that they were sufficiently clear, comprehensive, and fully captured the overall content of the data (Braun and Clarke, 2006; Nowell et al., 2017).

## Results

**Figure 1:**
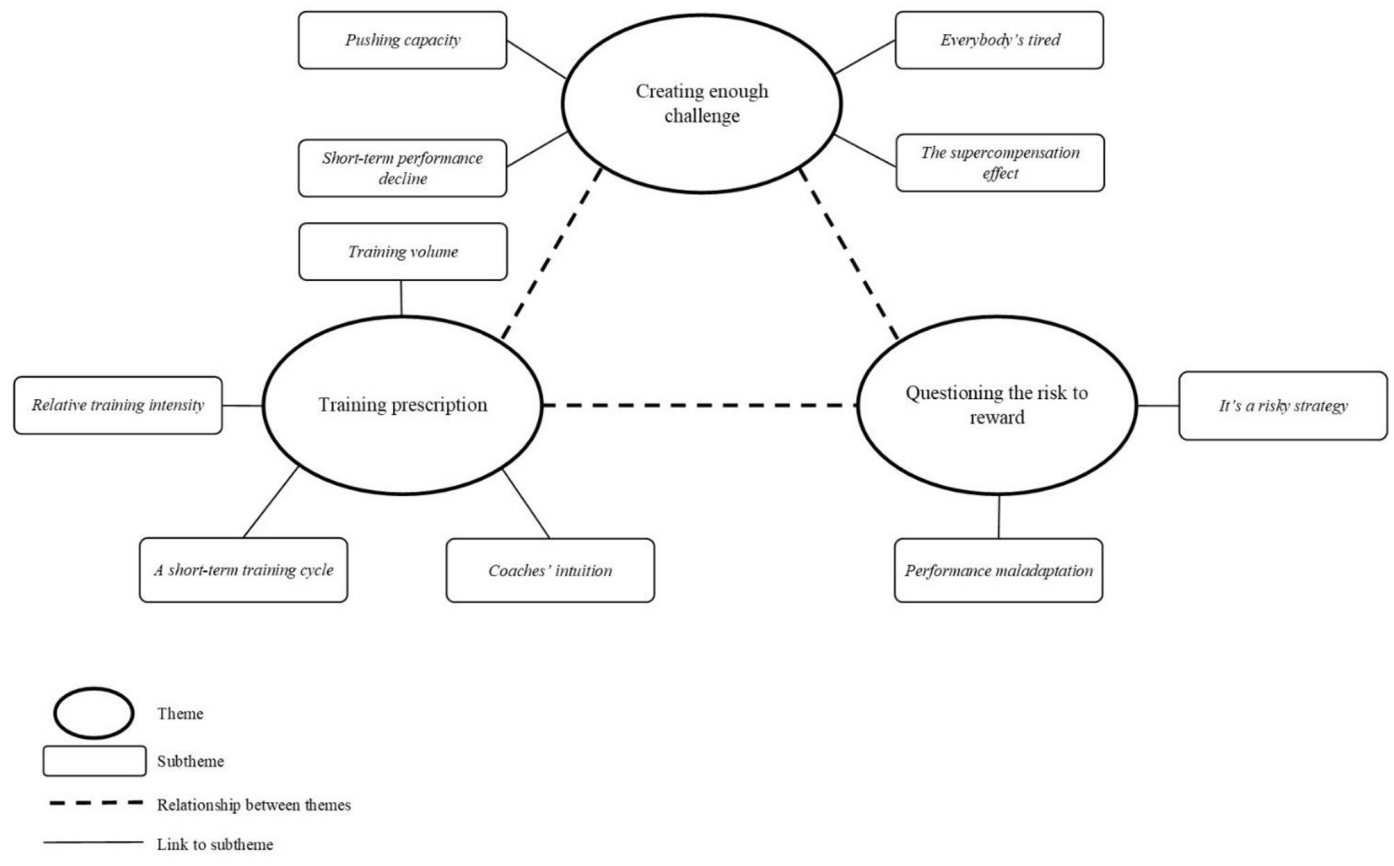
Schematic diagram of themes and subthemes.

The central concept of *planned overreaching* was organized into three themes to reflect the objectives of the research; *creating enough challenge, training prescription*, and *questioning the risk to reward*. Subthemes were developed to help manage the large amount of data and assist in the publicizing of information (see Figure 1, for a schematic representation of themes). To assist with the broadcasting of results, anonymised quotations have been used within the main report, attributed to the corresponding participant using the unique identification numbers presented in Table 1. Additional punctuation and parenthetical text have been added to direct quotations where required to improve comprehension.

### Creating Enough Challenge

In this theme, participants described POR as an opportunity to intentionally increase training demand to invoke the physiological adaptations that would result in positive performance improvement. Participants described how POR should “*overload”* and “*test”* the athlete, and that feeling “*beat-up”* and “*fatigued”* was an anticipated part of the training process. At times, symptoms of fatigue and muscle soreness were used “*as a marker”* to indicate successful training demand, and participants were “*not afraid”* of “*testing”* and pushing athletes “*hard.”* Participants described that whilst it was normal for athletic performance to decrease during, and in the days or weeks following POR, the end goal was to observe performance improvement above initial baseline, or “*supercompensation.”* The terms “*impact week,” “super-impact cycle,”* and “*red week”* were used colloquially to describe POR.

#### Pushing Capacity

Participants described the increase in training demand during POR as the “*driver”* of physiological adaptation and an opportunity to “*create enough challenge*.”

“*When you're on this cycle, you're really testing your body”* (11).

“*The purpose (of planned overreaching) is to push capacity. It's to force adaptation in the body by imposing greater demands than they've had previously, or recently”* (9).

Participants revealed that when undertaking POR, athletes would “*feel shit”* and “*beat up.”* But this was considered both procedural and anticipated. For this reason, it was common for participants to educate or “*warn”* their athletes in advance of the “*serious fatigue”* they would experience during and in the days following POR.

“*You ramp it up (training demand) to as much as they can deal with… to the point (that) it's about to crush them and kill them, and only then do you let them recover”* (9).

Athletes would be encouraged to “*smash it”* during POR. It was considered the point in the training programme where the athlete should “*push themselves to the limit”* and “*give it everything.”*

“*Can the lifter hack the training… the hard training? Do they really want it enough to be able to train hard enough to do these impact cycles? Can they handle pushing themselves right to the limit when they feel like they're going to be ill because they've really pushed themselves?”* (11).

#### Everybody's Tired

A consequence of undertaking high training demand during POR was an increase in “*soreness,”* feeling “*beat up”* and looking “*like crap.”* These were considered completely normal outcomes. In most cases, such manifestations were considered “*great markers”* to indicate that training demand was sufficiently challenging.

“*There are several times with athletes that they feel fucking shit and beat up going into the gym”* (2).

“*I will not wrap my athletes up in cotton wool. Everybody's tired every single day because that's the nature of the beast. But that's almost the aim of training. They're being loaded every other day. Sessions that make them vomit”* (8).

“*Fatigue is a great marker for ‘have we done enough to produce the reaction we want… or the adaptations we're hoping for?”'* (13).

#### Short-Term Performance Decline

A decrease in performance both during, and in the days and weeks that followed POR, was often described as just a “*part of training.”* Participants accepted that when undertaking POR, assessments of athletic performance would “*suffer.” T*his was viewed as evidence that POR was “*testing enough.”*

“*In the winter, guys will lift so much that they can't actually jump. Literally”* (10).

“*This is the interesting thing… I want to drive them down physiologically. They've got to feel shit for a long period of time… and I want to create so much stimulus that adaptation goes through the roof. I want that supercompensation”* (14).

“*They'll be grumpy, they'll be sore, and performance is really compromised”* (12).

“*The impact week is where they're working under some serious fatigue”* (1).

“*Along with that overreaching comes fatigue. I don't chase fatigue, but I'm aware it's going to happen… and (I) have no problem with that”* (2).

“*Fatigue is part of training… you can't hide from it”* (8).

“*They're three, four weeks into a fucking heavy block, they've done a lot of volume. I don't want them to be jumping as high. I don't believe that you should be fully or need to be fully recovered from every session. This idea that you need to optimize recovery, that you're fully recovered for every subsequent session? That's just not feasible”* (2).

#### The Supercompensation Effect

Participants revealed that the overall objective was to observe an improvement in performance relative to baseline after the initial period of performance decline. This was referred to as the “*compensation piece,” “rebound”* or “*supercompensation effect”* by participants. This effect was expected to occur within “*a couple of days”* to “*4 or 5, weeks”* after completion of the POR training cycle.

“*If they recuperate properly, they find (that) they've improved… and then they understand that's what it takes to be a top athlete”* (11).

“*…and then they wouldn't touch the gym at all, and they would get this huge kind of overshoot… the big compensation piece at the other end”* (10).

### Training Prescription

In this theme, participants described how they manipulated and organized training variables during POR. Most participants favored a combination of increased daily/weekly training volume with high relative training intensity to elicit the stimulus necessary to invoke the physiological adaptations necessary to observe a meaningful improvement in performance. POR was typically prescribed for a duration of 7–14 days (maximum 3 weeks) and performed several (5–8) weeks before competition.

#### Training Volume

Most participants revealed that training volume was the main variable by which POR was achieved. Such changes in training volume were facilitated through an increased number of training sessions per week, or through multiple training sessions per day. Increases in volume “*varied by athlete”* but would typically be “*ramped up”* or “*doubled”* to limit recovery between bouts and to “*train under fatigue from the session the previous day.”*

“*Volume is the stimulus and load is the consideration. It's always volume that I'll manage or manipulate during the overreach”* (9).

“*This is where we look to accumulate more volume”* (2).

“*On impact week we might increase the number of sessions from three or four to five or six. We might have multiple sessions per day… am and pm”* (9).

#### Relative Training Intensity

For some participants, an increase in relative training intensity was considered just as important as training volume to elicit the necessary increase in training demand. For one participant, it was an increase in relative training intensity, increased independent of volume, that provided the stimulus during POR.

“*I mean, load is probably more important… not so much volume. I'm really looking at quality over quantity… volume is actually driven more toward mediocrity to be honest”* (14).

For many participants though, it was the concomitant increase in training intensity and training volume that provided the “*unique feature”* of POR.

“*There's always got to be a point in training which is high-volume and high-intensity for you to elicit the right response”* (6).

“*If you're going for maximum volume, you're working up into the 90-95% intensity range… and working up to 100% maximum on your volume”* (11).

“*In the impact weeks, we'll do slightly heavier percentages, maybe for more sets”* (9).

#### A Short-Term Training Cycle

Participants rarely prescribed POR for periods of more than 2 weeks. For most, a 7–14 day training cycle was preferred, however, the specific number of training sessions within that period was dictated by individual athlete “*tolerance”* and response, varying from “*every other day”* to “*multiple sessions per day.”*

“*The length of the overreach will tend to be eight sessions over fourteen days”* (3).

“*They'd do like a ten-day or two-week block… smashing it for two or three weeks in the gym”* (12).

Importantly, POR was only implemented several weeks before a competition, to leave sufficient time for recovery and adaptation.

“*I would be setting that specific block probably 8 to 4 weeks out (from competition)”* (6).

How frequently POR was applied throughout the overall training programme was dictated by competition schedule. Additionally, the number of POR cycles completed within a training year was also determined by the athlete's previous experience of high training demand and their subsequent response as “*some athletes can tolerate more training and others can't.”*

“*I would say two (overreach) cycles in a twelve-week block. No more”* (11).

“*My go-to would be every four to six weeks in a ten to twelve-week phase. On the run-up to the competition, you might have two to three impact weeks”* (9).

For some, POR was a training tool used “*sparingly,”* reserved only for preparation for more important competitions or “*serious blocks”* of training.

“*We don't (overreach) often. It just depends on the importance of the competition”* (3).

#### Coaches' Intuition

Participants conceptualized POR as a flexible and individualized aspect of training, relying on their “*intuition”* and “*the art of coaching”* to guide the way that POR was organized and prescribed. Whilst there was congruence between participants in the overall objective of POR (to acheive FOR timed relative to competition), the precise strategy, magnitude and duration of a POR cycle was a highly individualized process, conducted using tacit knowledge and previous experience rather than reliance on rigid programming structure or objective assessment and monitoring.

“*Obviously a part of (planned overreaching) is actually having the intelligence to know when to step back when you need to step back and step forward when you need to step forward”* (14).

“*These are the types of things you might try: “let's do a block of high-intensity, low volume work (and) see how you respond. Next time. Let's do moderate, moderate” … and you do this for serious blocks to see what system the athlete seems to respond best (to) in terms of increases in overall strength”* (2).

### Questioning the Risk to Reward

In this theme, participants described the risks associated with POR: injury, “*burnout”* and/or “*overtraining.”* Participants explained that a positive performance adaptation (FOR) was not always guaranteed when undertaking POR: “*some people respond really well… some people break down completely.”* Moreover, a lack of knowledge, understanding, time and/or confidence resulted in some participants choosing to avoid using POR altogether in favor of “*less risky”* training methods.

“*I'm always cautious. The fact is, it's like jumping two-footed into a swimming pool”* (14).

“*I don't think it's worth pushing an athlete when they are failing to extremes. I've been coached like that. And I got very injured. I'm still dealing with the effects today”* (8).

#### It's a Risky Strategy

Some participants chose to avoid prescribing POR due to a lack of knowledge and/or confidence in their ability to organize training with “*precision”* in a way that elicited a positive performance outcome and avoided maladaptation. Participants described the difficulty in “*hitting the sweet spot.”* This was attributed to (1) the highly individualized athlete response to POR and (2) a lack of effective and reliable monitoring tools to proactively assess when training demand was sufficient to elicit the desired effect.

“*You know, overreaching is a risky strategy”* (6).

“*(Planned overreaching) is not really the kind of strategy that I would do to try and get performance gain. I probably just don't know enough about it… I think you need to be on the ball full time, doing omega waves [heart rate variability] every day, you know, looking at monitoring the sleep, monitoring the nutrition, calorie intake, all that kind of stuff to be able to start understanding that stuff”* (10).

“*I'm always questioning the risk to reward outlook on it… If they were a full-time professional, I think I'd have a lot more time to monitor them and a lot more time to actually go ‘let's get a little bit deeper on this”'* (14).

“*Some people respond really well to high-intensity work, high volume work (where) they're doing lots of doubles, triples. Some people break down and don't respond well to that”* (2).

For one participant, limited in-person coaching contact and emphasis on remote/online coaching was provided as a justification for avoiding POR.

“*You have to overreach with such precision. If I'm working with somebody who I don't see all the time and they're doing it remotely, I don't feel confident enough in my ability to be precise enough with the programming to get that exactly right. I don't think I can. I'm not going to lie to them (the athlete). I say, well, if you do this exactly perfectly, you can be supercompensated in a week? Nah.”* (5).

At times, an element of “*hope”* was required when undertaking POR, particularly when performance changes need to be timed accurately relative to competition.

“*Ultimately you'd ‘hope' that if the overreach is correct then the physical element should (be) supercompensated by the time they get into competition”* (3).

#### Performance Maladaptation

POR was considered by some participants to increase the risk of musculoskeletal injury, attributed to the combined effects of reduced coordination (caused by an increase in fatigue) and insufficient recovery between training bouts.

“*You're putting the athlete at risk (during overreaching). If you put them to that stage of fatigue through the gym work, then they're just neurally… they're just not coordinated. And when they're doing the running and the jumping work, they're far more at risk of injury at that point”* (10).

“*What's interesting is sometimes they continue to get worse rather than rebound after”* (1).

For one participant, undertaking periods of high-volume training combined with high-intensity training might result in “*burnout”* (a term that they used synonymous to injury).

“*Powerlifting athletes often do periods of quite high-intensity coupled with quite high-volumes… and these (can) generally lead to drastic improvements in strength in the short-term, but all the time end up in, the term is… “burnout”. They just end up with loading issues and injury… and actually, athletes will generally phrase (it) as “got burnt out,” but really it's just, ‘got injured”'* (2).

At times, participants alluded to a “*fine line”* between insufficient, optimal and excessive training demand (“*banging the athlete up a little)*. However, participants were generally dismissive of the risk of long-term performance decline indicative of OTS.

“*Yeah, it's hard to pick a point where it's not worked… or I've ‘overtrained' them”* (3).

“*I haven't had anyone feeling ‘pretty broken' yet”* (14).

In many cases, the risk attributed to miscalculation of training during POR was not in the potential maladaptive response caused by excessive training demand, but by a lack of performance improvement caused by insufficient training demand.

“*I'm always questioning the risk reward outlook on it. Am I putting enough risk in that programme to get the desired reward?”* (14).

“*Worst case scenario hurt the person or just bang them up for a little while and they don't actually super compensate… or you undershoot it and then you just essentially didn't work hard enough… and those outcomes are much more likely than exactly hitting the sweet spot”* (5).

## Discussion

The aim of this study was to examine high-performance strength coaches' perceptions and experiences of POR and to determine how coaches conceptualize POR as a training tool. We identified three themes that provide important practical information regarding POR from the perspective of the strength coach: *creating enough challenge, training prescription*, and *questioning the risk to reward*. Findings demonstrate that POR is typically implemented in the weeks preceding competition, achieved through a deliberate and sometimes considerable increase in training volume and/or training intensity, for a period of 7–14 days. A short-term decrease in performance capacity, both during and in the days to weeks following training is an anticipated consequence of POR. Moreover, when combined with symptoms of fatigue, soreness, and reduced motivation to train, short-term performance decline was used as a benchmark to confirm sufficiently challenging training demand. Some participants chose to avoid prescribing POR due to a lack of knowledge, confidence, and/or increased risk of training maladaptation (i.e., musculoskeletal injury). Participants approached the design of POR in an intuitive and individualized way, relying on both tacit knowledge and previous experience to inform programming decisions to achieve the best outcome. Additional findings note the disconnect between POR conceptualized by the high-performance strength coach and the POR protocols used in previous well-controlled research studies.

### What Did Coaches Consider the Objective of Planned Overreaching to Be?

Participants conceptualized POR as a tool to induce the physiological adaptations required to achieve a meaningful standard of performance improvement. POR was described as a point within the overall training programme where the athlete could be challenged with intense and frequent overloading of training. As such, participants anticipated that both during, and in the days that followed completion of POR, athletes would experience a relative decrease in physical performance. Additional symptoms of increased general fatigue, musculoskeletal soreness and negative mood were also to be expected, and used procedurally to verify that training was sufficiently challenging. Whilst the primary aim of POR was to observe an increase in athletic performance, participants accepted there was an inherent element of both “*hope”* and “*risk”* when undertaking POR, aware that even through carefully organized POR, either a lack of performance improvement (caused by insufficient training demand challenge) or maladaptation (caused by excessive and/or prolonged training demand) could occur.

POR has led to both improved sport specific and general measures of performance indicative of FOR (Häkkinen et al., 1989; Warren et al., 1992; Fry et al., 1993, 2000a; Pistilli et al., 2008; Bazyler et al., 2017; Suarez et al., 2019). However, POR has also resulted in training maladaptation indicative of NFOR (Fry et al., 1994c,d, 1998, 2006; Purdom et al., 2021). Within expert consensus, the response to overloading training has been described as a continuum, where FOR precedes NFOR and OTS manifests as an extension of NFOR if training persists/recovery is insufficient (Meeusen et al., 2013). However, the response to recurrent overloading training (as observed during POR) is multifactorial and complex, and therefore is likely to be an oversimplification (Kataoka et al., 2022). This is reflected in the findings from this research, as several participants described successful POR (i.e., results in FOR) as a flexible and intuitive process guided by tacit knowledge and experience, as opposed to a rigid approach to programming or reliance on prognostic assessment to guide decision making.

### How Did Coaches Organize and Manipulate Training During Planned Overreaching?

Participants implemented POR through an increase in training volume (achieved through increased daily or weekly training) and/or relative training intensity. In many cases, it was the concomitant increase in volume and relative training intensity that provided the unique stimulus necessary to invoke physiological adaptation and subsequent performance improvement. POR was considered a short-term, “impact” cycle, often prescribed for periods of 7–14 days, used sparingly across the competition schedule. No single best practice method of POR was revealed during this research. Instead, POR was individualized to the athlete in an intuitive way, suggesting that the coach's experience is an important factor in successful POR, and not just the increase in training demand. Consequently, participants rarely alluded to detailed changes to specific intensities, exercise selection or total volume.

In contrast to the intuitive, instinctive approach to POR revealed by participants of this research, previous studies have used well-controlled prescriptive high-volume (Fatouros et al., 2006; Wilson et al., 2013; Lowery et al., 2016) and high-intensity (Fry et al., 1994a,c,d, 1998, 2000b, 2006; Sharp and Pearson, 2010; Nicoll et al., 2016; Sterczala et al., 2017) resistance exercise POR protocols to investigate potential diagnostic markers of FOR and NFOR/OTS. Such protocols have incorporated either single exercise (typically the barbell back squat) (Fry et al., 1994a,c,d, 1998, 2000b, 2006; Nicoll et al., 2016; Sterczala et al., 2017) and multiple exercises (Ratamess et al., 2003; Volek et al., 2004; Fatouros et al., 2006; Kraemer et al., 2006; Sharp and Pearson, 2010; Lowery et al., 2016; Drake et al., 2017), and both traditional strength-based exercises (squat variations, pulls and presses) and sport-specific exercises (snatch, clean and jerk, throwing drills) (Fry et al., 1993, 2000a; Hartman et al., 2007; Bazyler et al., 2017) have been selected. Overall, the number of studies reporting either no performance maladaptation (i.e., return to baseline) or performance improvement outweigh those that have observed NFOR/OTS (Bell et al., 2020; Grandou et al., 2020b). Taken as a body of literature, these protocols indicate which types of training might increase susceptibility to NFOR/OTS, but due to methodological heterogeneity makes comparisons between research studies difficult. Moreover, an absence of follow-up performance assessments, and failure to reliably induce physiological, biomechanical, or hormonal alterations has led to a lack of reliable assessment for the prognostic identification of NFOR/OTS. To date, no standardized POR protocol exists within the literature, however, the development of a single best practice POR protocol might be misplaced given the complexity of high-performance strength training and variability in response to POR.

Participants of this research developed POR with the objective to enhance the physiological adaptations required to achieve a meaningful standard of performance improvement. Conversely, many of the protocols used in previous research studies have been designed not to *improve* physical performance, but to *induce* a state of training maladaptation for the purpose of elucidating diagnostic information. Consequently, current understanding of NFOR/OTS is limited (and likely insufficient) due to incongruence between the mechanisms being explored during previous research and their intended outcome within a practical context. Whilst the number of POR studies reporting return to baseline or FOR outweigh those that have observed NFOR or OTS (Bell et al., 2020; Grandou et al., 2020b), it is unsurprising that some types of POR are likely to increase the susceptibility to maladaptation, such as those including repeated use of daily high volume maximal loads with low exercise variation (Bell et al., 2020; Grandou et al., 2020b). For example, the most commonly-prescribed protocol used in the literature (10 x 1 repetition at 100% one-repetition maximum squat machine for 14 successive days) was developed as an “*overtraining protocol”* to identify potential markers of training maladaptation, and has reported consistent performance decrements indicative of NFOR or the OTS (Fry et al., 1994c,d, 1998, 2006). However, based on what participants of our research reveal about POR, this protocol does not reflect the typical approach to POR used within a high-performance strength sport training environment to enhance performance. Whilst research designed to induce training maladaptation does provide important contextual information that a dose-response “threshold” might exist (as well as possible markers to identify maladaptation), caution must be given when transferring those findings into the practical training environment, where the design and prescription of POR is more intuitive and flexible.

Previous research has indicated that there is not only variability in the physiological response to different approaches to POR training (e.g., high-volume vs. high-intensity) (Fry and Kraemer, 1997; Bell et al., 2020; Grandou et al., 2020b), but that differences might also occur in a group of individual athletes undertaking the *same* training protocol. These differences are likely to be modulated by multiple factors including genotype (Clarkson et al., 2005), sex differences (Hunter, 2016) muscle fiber typology (Bellinger et al., 2020; Lievens et al., 2020), age, and biological maturation (Moran et al., 2017). Additional factors such as level of competition/status (elite vs. non-elite) (Kreher and Schwartz, 2012) and the athlete's “stress capacity” (i.e., the ability to tolerate the combined effects of training and non-training stressors) (Kenttä and Hassmén, 1998; Stults-Kolehmainen and Bartholomew, 2012) are also likely to play a role in the response to POR. It is therefore completely plausible that some athletes will be more predisposed to the effects of training maladaptation during periods of POR, and therefore POR would need to be individualized to the athlete to achieve an optimal performance outcome. This might, in part, reflect why participants of this study approached the implementation of POR intuitively, and on an athlete-by-athlete basis. Moreover, the high inter-individual and exercise-specific variability in response to POR may in part explain why there is a lack of reliable markers and measures able to detect NFOR/OTS. The absence of a single, reliable marker to detect training maladaptation is unsurprising when this is considered, and future research should explore the inter-individual response to POR to further understanding in this field.

### What Did Coaches Consider the Potential Risks of Planned Overreaching to Be?

Participants of this research were largely unconcerned about the risk of NFOR or OTS caused by POR. However, some did consider POR to be a strategy that *could* result in musculoskeletal issues (i.e., injury) if the demands of training were miscalculated. For others, the risk involved in undertaking POR was more related to providing an insufficient training demand (and therefore a lack of challenge) that would not elicit a positive response; a concern attributed to a lack of knowledge and/or confidence in prescribing effective POR.

Previous research has indicated that injury prevalence in strength sports such as powerlifting and weightlifting is relatively low, especially when compared to contact sports (Keogh and Winwood, 2017). Additionally, the prevalence of musculoskeletal injury reported in the strength sport literature is low (Bell et al., 2020; Grandou et al., 2020b), with only a single study reporting musculoskeletal injury as a concomitant symptom of maladaptation following POR (Fry et al., 2001). Conversely, high-performance strength coaches consider musculoskeletal injury to be the most common symptom of NFOR/OTS (Bell et al., 2021), and competitive strength athletes who have experienced an unexplained decrease in performance report musculoskeletal issues (i.e., aches and pains) as the most common symptom of maladaptation (Grandou et al., 2020a). It is worth noting that musculoskeletal issues have been most frequently reported where the decrease in performance lasted <1 week to 1 month, but not >1 month, suggesting aches and pains are more indicative of acute maladaptation and not actually NFOR/OTS. Whilst the general consensus in the literature is that repeated high-intensity resistance exercise might increase the risk of musculoskeletal and musculotendinous injury, injury epidemiology is multifactorial in nature and differs by both proportional injury rate and severity across strength sports (Keogh and Winwood, 2017). There is currently a lack of research investigating the onset of injuries, the manner in which injuries affect training, and the necessary recovery required after musculoskeletal injury in competitive strength sports (Keogh and Winwood, 2017).

## Practical Applications

The information provided by participants of this research better contextualizes POR from the perspective of the strength coach and demonstrates both the intuitive and individualized nature of high-performance strength sport training. Additionally, this research highlights the potential dichotomy between POR protocols used in practice, and those used for the sole purpose of diagnostic identification of FOR and NFOR/OTS within the literature. Expert coaches exhibit characteristics of knowledge, talent, pedagogy, and perseverance, as well as the procedural ability to transfer information rationally using experience and intelligence (Dorgo, 2009). There is also a high level of intuition in identifying and solving programming errors in an instinctive way (LaPlaca and Schempp, 2020). However, such tacit knowledge is difficult to articulate, and coaches are not always aware of their decision making; rather, it is guided by intuition, instinct and experience rather than theory or pedagogy (Nash and Collins, 2006). Previous research has suggested an ever-increasing body of empirical research dedicated to optimizing athlete training practices, yet there remains a considerable gap between science and best practice (Haugen, 2021; Haugen et al., 2021). Participants of this research described POR as a multifactorial and individualized process, and therefore a complex aspect of sports performance support (Greenberg and Clubb, 2021). Therefore, it appears more appropriate to consider the development of “good” training practices and guidelines rather than a single best practice approach to POR, as multiple solutions appear to exist in the context of POR within the “real world” of strength sports, illustrated by the different approaches described in this research (e.g., high training volume vs. high intensity). Such guidelines would provide a framework of decision-making for coaches who wish to attempt POR with their athletes, but at the same time allow flexibility based on marked inter-individual variability in the response to periods of high training demand. As such, the preceding recommendations have been developed to assist both coaches and sport scientists in the development of POR protocols for research and/or training purposes.

## Strengths and Limitations

Participants of this study provided important contextual information relating to their perceptions and experiences of POR within the strength sport coaching community. This information can be used by coaches to further their understanding about the conceptualization and implementation of POR in a real-world setting. However, this study also provides important guidance for sport scientists who intend to design POR protocols that reflect the multidimensional and complex nature of training practices for the purpose of scientific investigation.

The qualitative nature of this research facilitated a reflexive but systematic approach. Verification strategies involved during the analysis of interviews such as development of an audit trail through maintenance of a code book, research team standardization checkpoints, and member checking enhanced the credibility and trustworthiness of the results. Findings should serve not only as a catalyst for further investigation into the nuances of optimal POR (i.e., leading to improved performance relative to initial baseline), but also as an opportunity for collaboration between coaches and sport scientists to improve overall understanding in this domain.

Whilst this study offers new insight into POR, it is recognized that methodological limitations do exist. The participant pool for this research derived from strength coaches within a homogeneous community. Future research might benefit from the perspectives of a broader scope of coaches (i.e., those involved in sports where strength training is an important, but not the only, component of the overall training programme such as intermittent sports or those involving concurrent training methods). The recruitment strategy used for this research followed an opportunity, snowball approach, focused primarily on social media. Whilst this provided an efficient and fair approach to recruitment, it might also have biased participants who regularly accessed social media, whilst simultaneously excluding those who met the inclusion criteria, but were unaware of the opportunity to participate in the research.

## Data Availability Statement

The original contributions presented in the study are included in the article/Supplementary Materials, further inquiries can be directed to the corresponding author.

## Ethics Statement

The studies involving human participants were reviewed and approved by Sheffield Hallam University. The participants provided their written informed consent to participate in this study.

## Author Contributions

LB, AR, TM-W, and DR contributed to the conceptualization of the study methodology. LB developed the original draft preparation. All authors were involved in review and editing of the manuscript prior to submission and have approved and agreed to the published version of the manuscript.

## Conflict of Interest

The authors declare that the research was conducted in the absence of any commercial or financial relationships that could be construed as a potential conflict of interest.

## Publisher's Note

All claims expressed in this article are solely those of the authors and do not necessarily represent those of their affiliated organizations, or those of the publisher, the editors and the reviewers. Any product that may be evaluated in this article, or claim that may be made by its manufacturer, is not guaranteed or endorsed by the publisher.
