# Erratum: “I Want to Create So Much Stimulus That Adaptation Goes Through the Roof”: High-Performance Strength Coaches' Perceptions of Planned Overreaching

**DOI:** 10.3389/fspor.2022.937588

**Published:** 2022-05-20

**Authors:** Frontiers Production Office

**Affiliations:** Frontiers Media SA, Lausanne, Switzerland

**Keywords:** overreaching, functional overreaching, overtraining, strength sports, strength training

Due to a production error, participant numbers in the text were displayed incorrectly. A correction has been made to the section **Material and Methods**, subsection **Participants and Sampling**. The corrected text appears below:

“weightlifting; *n* = 5, powerlifting; *n* = 4, sprinting; *n* = 2, throws; *n* = 2, jumps; *n* = 1”

The publisher apologizes for this mistake. The original article has been updated.

