# Supplementary material for: “I Want to Create So Much Stimulus That Adaptation Goes Through the Roof”: High-Performance Strength Coaches' Perceptions of Planned Overreaching
Source: Front Sports Act Living. 2022 May 2;4:893581. doi: 10.3389/fspor.2022.893581 (PMC9108365; doi:10.3389/fspor.2022.893581)
Supplement: Supplementary file 1 [file Table_1.pdf]

Appendix 1: Semi-structured Interview Guide

**Introductory questions/background information**	1. *Tell me a bit about yourself and what sport(s) you are involved in?*<br>*To cover:*<br>- *Country of residence*<br>- *Experience (level of athlete, duration in years)*<br>- *Highest academic qualification*<br>- *Professional qualifications*
**Central interview questions**	***General approaches to strength sport***<br><br>2. *Would you consider yourself to be involved within elite-level strength sport? If so, how would you define 'elite' athletes in your sport?*<br>3. *Do you have a specific coaching philosophy? What governs this?*<br>4. *Do you have a favoured or 'go to' periodized approach to training?*<br><br>***Monitoring***<br><br>5. *Do you monitor athlete progress? If so, how?*<br>6. *What tests/measures do you use to monitor progress and/or identify fatigue? (do these differ based on purpose?)*<br>7. *Do you find that athletes experience training-related fatigue often? When?*<br>8. *What do you think contributes to general fatigue in your athletes?*<br>9. *From your experience, what types of non-training stressors do you think contribute to athlete fatigue (if any)?*<br>10. *Would you adapt training if you identified fatigue? If so, how?*<br><br>***Programming***<br><br>11. *Do you use periods of concentrated loading (high training demand) weeks to create performance changes? If so, when, how might that look, and what variables do you alter to elicit those changes?*<br>12. *What are your thoughts relating to high training demand weeks as a training tool (risk/reward)?*<br>13. *(Return back to fatigue questions) Does your approach to monitoring/testing alter based on if an athlete if undertaking intentional periods of high training demand?*<br>14. *How would you define overtraining?*<br>15. *Have you observed or experienced overtraining in your sport (or heard of other coaches/athletes that have)?*<br>16. *Do you think overtraining exists within strength sports?*<br>17. *What symptoms would you expect to see in an overtrained athlete?*<br>18. *How long do you think it would take an overtrained athlete to fully recover?*<br>19. *Do you have any other additional information you would like to add relating to perceptions of overtraining?*
**Closing questions**	20. *Before we finish, is there anything you would like to elaborate on or add to the discussion?*
